# Media use degree and depression: A latent profile analysis from Chinese residents

**DOI:** 10.3389/fpsyg.2022.1070774

**Published:** 2023-01-17

**Authors:** Fangmin Gong, Pei Yi, Lian Yu, Siyuan Fan, Guangze Gao, Yile Jin, Leixiao Zeng, Yang Li, Zheng Feei Ma

**Affiliations:** ^1^College of Literature and Journalism Communication, Jishou University, Jishou, China; ^2^College of Public Health, Xi’an Jiaotong University Health Science Center, Xi'an, China; ^3^Department of Preventive Medicine, Yanjing Medical College, Capital Medical University, Beijing, China; ^4^Tongliao Clinical College, Inner Mongolia Medical University, Tongliao, China; ^5^Baotou Clinical College, Inner Mongolia Medical University, Baotou, China; ^6^College of Journalism and Communication, Renmin University of China, Beijing, China; ^7^College of Communication and Art Design, University of Shanghai for Science and Technology, Shanghai, China; ^8^Centre for Public Health and Wellbeing, School of Health and Social Wellbeing, College of Health, Science and Society, University of the West of England, Bristol, United Kingdom

**Keywords:** media use, depression, Big Five personality, cross-sectional study, latent profile analysis

## Abstract

**Background:**

Previous studies have emphasized the media as an essential channel for understanding information about depression. However, they have not divided groups according to the degree of media use to study their differences in depression. Therefore, this study aims to explore the influence of media use on depression and the influencing factors of depression in people with different media use degrees.

**Methods:**

Based on seven items related to media use, a total of 11, 031 respondents were categorized by the frequency of media use using latent profile analysis (LPA). Secondly, multiple linear regression analyzes were conducted to analyze the effects of depression in people with different degrees of media use. Finally, factors influencing depression among people with different degrees of media use were explored separately.

**Results:**

All respondents were classified into three groups: media use low-frequency (9.7%), media use general (67.1%), and media use high-frequency (23.2%). Compared with media use general group, media use low-frequency (*β* = 0.019, *p* = 0.044) and media use high-frequency (*β* = 0.238, *p* < 0.001) groups are significantly associated with depression. The factors influencing depression in the population differed between media use low-frequency, media use general, and media use high-frequency groups.

**Conclusion:**

The government and the appropriate departments should develop targeted strategies for improving the overall health status of people with different media use degrees.

## 1. Introduction

Depression is one of the most common mental disorders. Depression is characterized by low mood and reduced concentration, and restless sleep (Nimrod, 2013). More than 300 million people worldwide suffer from depression, which leads to self-harm or suicide, eventually leading to death (World Health Organization, 2016). Depression usually lasts for a long time, and its burden exceeds that of depression itself (Lepine and Briley, 2011), seriously damaging the quality of life of the public (Daly et al., 2010). In many countries, the prevalence of depression is rising rapidly (Fleming et al., 2014; Holling et al., 2014; von Soest and Wichstrom, 2014). The current COVID-19 (New Coronary Pneumonia) outbreak has led to rising public depression (Rodriguez-Hidalgo et al., 2020). Research findings in China showed that at the beginning of the outbreak, there were increasing symptoms of anxiety, pressure, and depression in public (Zhong et al., 2021). Suicidal thoughts manifest themselves in China’s general population due to the uncertainty of the epidemic situation and unpredictable consequences (Shi et al., 2021). Many scholars have focused their research on depression to reduce the burden of depression (Fujise et al., 2016; Assari, 2017; Shensa et al., 2017).

The media is a channel for the public to learn about various health issues (Petersen, 2001; Seale, 2003). Broadly, the media includes all kinds of media, such as newspapers, magazines, radio, TV, and mobile phones. Previous studies have shown that the use of media, such as social media, video games, TV, and movies, is related to the development of public depression (Kross et al., 2013; Bickham et al., 2015; Liu et al., 2016; Holfeld and Sukhawathanakul, 2017). It has been found that excessive use of social media by the public will lead to more severe depression during COVID-19 (Zhong et al., 2021). Frost and Rickwood concluded that media use has both positive and negative effects on depression (Frost and Rickwood, 2017). Some scholars, however, argue that there is no apparent connection between media use and depression (Jelenchick et al., 2013; Banjanin et al., 2015; Nesi et al., 2017). Other scholars believe that the relationship between media use and depression is complicated, and there is no clear conclusion that needs further study (Blease, 2015; Seabrook et al., 2016). In order to answer the question of what factors affect public depression, it is necessary to investigate whether there is a significant relationship between media use and depression.

Several studies have shown that variables associated with social demography can influence public depression, such as gender, marital status, education, occupation (Andersen et al., 2011). Various factors can contribute to depression, including pressure, Big Five personality, and social support (Neese et al., 2013; Kayiş et al., 2016; Bukhari and Afzal, 2017). For instance, a person who feels more pressure is more likely to suffer from depression (Dejonckheere et al., 2017). According to the Big Five personality traits, extraversion, agreeableness, conscientiousness, and openness were negatively associated with depressive symptoms, while neuroticism was positively associated (Nikcevic et al., 2021). Besides, social support is an important predictor of depression (Alsubaie et al., 2019). Depression is influenced by family and friend support (Chang et al., 2018). Some studies showed that people with little or no support from friends are more likely to experience depression (Chen et al., 2020). These factors were used as control variables to analyze how the degree of media use correlates with depression.

Furthermore, previous research has demonstrated a significant relationship between media use and social support, pressure, Big Five personality, and depression (Cellini et al., 2020; Meshi and Ellithorpe, 2021; Przepiorka et al., 2021). Using the internet excessively can lead to low social support, resulting in depression (He et al., 2014). Stressed students may experience depression and use the internet excessively to relieve their low mood, insomnia, fear, and despair (Ranjan et al., 2021). The relationship between media use and depression may be moderated by extraversion in the Big Five personality traits (Weiß et al., 2022). Moreover, the effect of social networking sites on depression may be moderated by demographic factors such as age and gender (Pfeil et al., 2009; Steers et al., 2014; Frison and Eggermont, 2016). Additionally, this study will examine depression in populations with different degrees of media use based on demographic variables, pressure levels, social support, and Big Five personality traits. For evaluating the use of a single media, most studies used small samples, such as those using Facebook, or focused on young people (Koc and Gulyagci, 2013; Schou Andreassen et al., 2016). Therefore, based on a Chinese sample, this study first classified people with different media use degrees by latent profile analysis (LPA), aiming to explore the relationship between people with various media use degrees and depression. Then, multiple linear regression analysis is used to determine the factors that influence the depression possibility of the public. These findings intend to provide suggestions for clinical and public health interventions.

## 2. Methods

### 2.1. Ethics statement

The study protocol was approved by the Institutional Review Committee of Jinan University (JNUKY - 2021-018), Guangdong, China. All participants voluntarily participate in the survey and are required to sign an informed consent form.

### 2.2. Research object

Inclusion criteria: ① Aged ≥ 12 years; ② Members of the public with the nationality of the People’s Republic of China; ③ Public with permanent residence in China (Annual departure time ≤ 1 month); ④ Voluntary participation in the study and completion of the informed consent form; ⑤ Members of the public who can complete the web-based questionnaire on their own or with the help of the investigator; ⑥ Understanding the meaning expressed in each entry of the questionnaire.

Exclusion criteria: ① those who are confused mentally abnormal or affected by cognitive impairment; ② those who are participating in other similar research projects; ③ those who are unwilling to cooperate.

### 2.3. Survey method

This survey was conducted between July 10, 2021, and September 15, 2021.

Multi-stage sampling method was adopted, starting from the capitals of 23 provinces, five autonomous regions, and four municipalities directly under the Central Government of China. Then, using the random number table method, 2–6 cities in each province and non-capital prefecture-level administrative region of an autonomous region were selected for a total of 120 cities, and at least one investigator or investigation team (≤ 10 people) was openly recruited. Based on the “7th National Population Census in 2021” results, a quota sample (quota attributes of gender, age, and urban–rural distribution) was taken from the residents of the 120 cities to make it broadly consistent with the demographic characteristics of the country. Each city needed to recruit at least one investigator or team. Each investigator was responsible for collecting 30–90 questionnaires, and each investigation team was responsible for collecting 100–200 questionnaires.

### 2.4. Instruments

#### 2.4.1. Basic information survey

The questionnaire covered basic demographic information (such as gender, age, education level, etc.), media use, social support, pressure, Big Five personality, and depression. Among them, media use, and pressure were self-designed scales, while social support, depression, and Big Five personality were international general scales.

#### 2.4.2. Self-made media usage scale

The self-made media usage scale was used to measure the type and frequency of Media usage. After a scientific and comprehensive review of books and literature, members of the research team designed the questionnaire (Frederick et al., 2017; den Hamer et al., 2017), and on June 7, June 11, June 15, June 18, July 3, and July 8, 2021, expert (all with senior titles and regional representation) were consulted and discussed to ensure that the questionnaire applies to all media-using populations. Seven items on the scale were used to know the frequency of exposure to 7 kinds of media: newspapers, magazines, radio, television, books (not textbooks), personal computers (including tablets), and smartphones. Each item was set with five options: Never use, Occasionally use (≤ 1 day/week), Sometimes use (2–3 days/week), Frequently use (4–5 days/week), and Almost every day (6–7 days/week), which were assigned to 1–5 in turn (never use = 1, almost every day = 5). The number of days that the measured person was exposed to various media in 1 week was used as the scoring basis, and the total score of each option was added as the scoring result, with a total score of 35 points. A higher score indicates that the subjects’ media use was higher. The Cronbach’s alpha of the scale was 0.70.

#### 2.4.3. 9-item patient health questionnaire

The 9-item Patient Health Questionnaire scale (Löwe et al., 2004) assessed depression based on the subject’s self-assessment scores over the past 2 weeks. Both had good reliability and validity in helping to diagnose depression and assessing the severity of symptoms. Seven items on the scale were used to set up four options “hardly ever,” “a few days,” “more than half,” and “almost every day.” Scores were assigned to 0–3 (almost none = 0). Each option had a total score of 27 points. The higher the score, the more depressed the tendency. The Cronbach’s alpha of the scale was 0.94.

#### 2.4.4. Self-made pressure scale

A self-made scale was used to measure the sense of self-pressure (Zhang et al., 2022). The scale consists of three questions, which mainly focus on the ability of individuals to cope with stress. It takes 2 weeks to 1 year to perceive and evaluate stress in life (including work and family). The score of each item is between 1 point and 6 points, and the total scores of the three items are added up as the scoring result. The total score ranges from 3 points to 18 points, and the higher the score, the more significant the sense of stress. Residents select scores to reflect their level of stress according to their actual situation. The Cronbach’s alpha of the scale was 0.86.

#### 2.4.5. 10-item Big Five inventory

The Big Five Personality Scale was used to measure personality traits (Gosling et al., 2003). The scale consists of 5 items: openness, agreeableness, neuroticism, conscientiousness, and extraversion, with each item containing two entries (10 entries in total). All questions on the scale are scored on a five-point Likert scale, and each subscale is scored out of 10, with five items being scored positively and five items being scored negatively. The higher the score, the more pronounced the personality trait. The Cronbach’s alpha of the scale was 0.94.

#### 2.4.6. Perceived social support scale

The PSSS was used to measure social support (Zimet et al., 1990). PSSS includes 12 items assessing emotional support from friends, family, and significant others. Every item had seven options ranging from “extremely disagree” to “extremely agree,” which were rated 1–7 in turn (extremely disagree = 1, extremely agree = 7). Each item was scored according to the degree of consent. Based on the scores of all items, a total score from 12 to 84 was calculated, indicating the degree of social support felt by an individual. The higher the score, the greater the degree of support. The Cronbach’s alpha of the scale was 0.96.

#### 2.4.7. Data analyzes

M ± SD expressed continuous variables, Chi-square (*x^2^*) tests were used for comparisons between groups, and categorical variables were expressed as frequencies. The potential profile of seven items used by media was analyzed by Mplus 8.3 software, the best model is determined mainly by Akaike Information Criteria (AIC), Bayesian Information Criteria (BIC), Adjusted Bayesian Information Criteria (aBIC), Entropy, Lomendell-Rubin (LMR), and Bootstrap Likelihood Ratio Test (BLRT). The smaller the value of AIC, BIC, and aBIC, the better the fit of the LPA to the data was. Entropy was between 0 and 1, and the closer to 1, the more accurate the classification. The significant difference between LMR and BLRT (*p* < 0.05) indicated that the k-type model was superior to the K-1 model. The number of categories in the model gradually increased from the initial model until the model with the best-fitting data was found. SPSS 26.0 software was used for stepwise regression analysis based on retaining the best category model. *p* < 0.05 (two-side) was used to indicate statistical significance.

## 3. Results

### 3.1. Analysis of potential profile of media use

**Figure 1:**
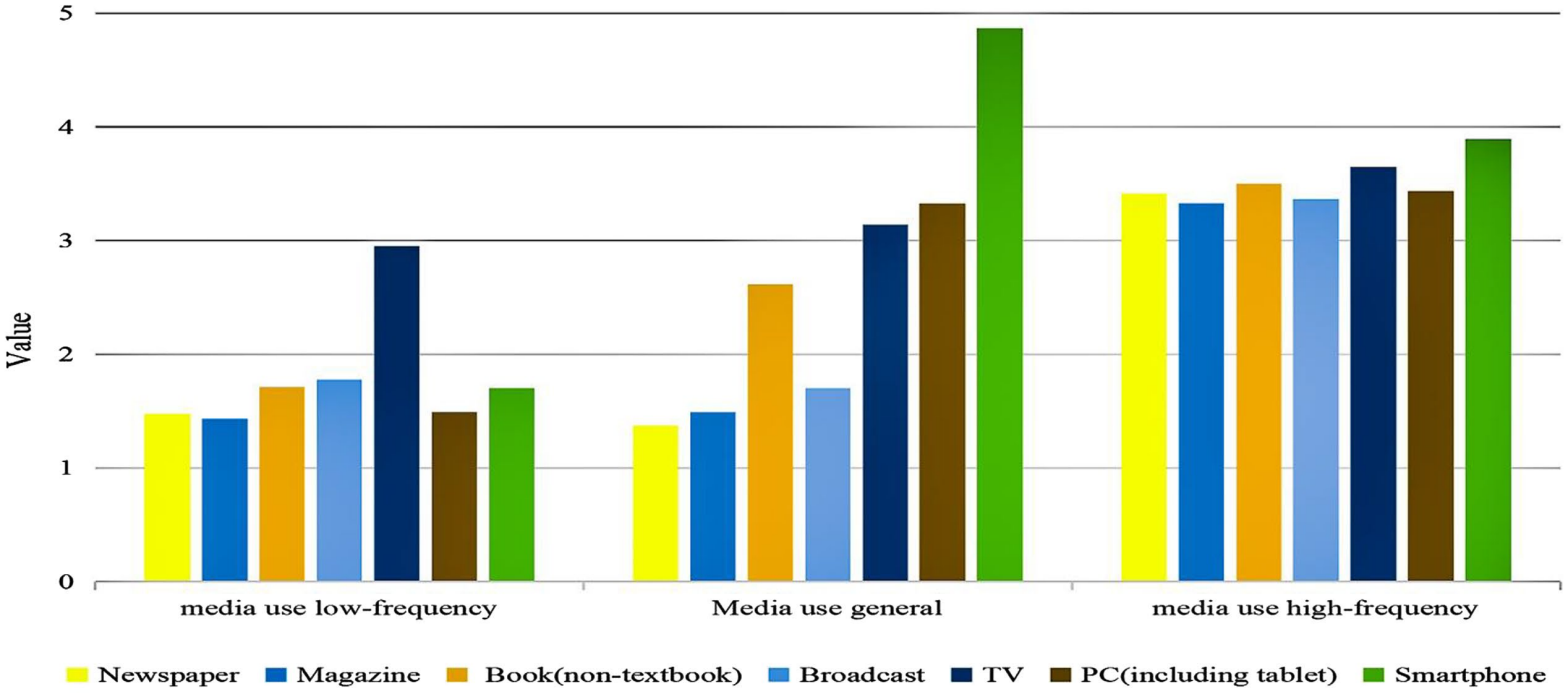
Profile of potential categories of media use.

We selected 1–6 latent profile models to analyze media use frequency. The results showed that the values of AIC, BIC, and aBIC decreased with the number of categories increasing. Two indicators of LMR and BLRT (*p* < 0.001) indicated that the models fit well for Class 2, 3, 4, and 5. The value of Entropy is closest to 1 for Class 4, followed by Class 3. The analysis was combined with the model diagrams of each category (Figure 1), and a classification model of three potential categories (C1, C2, C3) was finally chosen to classify the residents’ media use frequency. As shown in Table 1.

**Table 1:** Latent profile model indexes of fitting for media usage types.

s	K	AIC	BIC	aBIC	Entropy	LMR	BLRT	Class probability (%)
1	14	246944.918	247047.237	247002.746				1
2	22	230380.614	230541.400	230471.487	0.919	<0.001	<0.001	0.747/0.253
3	30	221958.644	222177.898	222082.562	0.948	<0.001	<0.001	0.097/0.672/0.231
4	38	216424.795	216702.517	216581.758	0.959	<0.001	<0.001	0.089/0.115/0.668/0.128
5	46	208110.241	208446.430	208300.248	0.943	<0.001	<0.001	0.298/0.207/0.262/0.134/0.098
6	54	207582.155	207976.812	207805.207	0.985	0.994	1.0000	0.449/0.080/0.080/0.239/0.055/0.098

Three potential categories differed obviously in their frequency means for the seven items of media use and showed different characteristics. C1 accounted for about 9.7% of the total number of subjects, and its score (12.515 ± 1.788) on each item was significantly lower than that of C2 and C3. Therefore, this category was named “media use low-frequency.” C2 accounted for approximately 67.1% of the total, and its score (18.504 ± 2.643) on each item was higher than C1 but lower than C3 and was therefore named “media use general.” C3 accounted for 23.2% of the total, and its score (24.571 ± 3.510) was significantly higher than C1 and C2. This category was therefore named “media use high-frequency.” The Probability of attribution for three potential types ranged from 0.95–0.96, indicating that the classification results of the three potential category models were highly reliable. As shown in Figure 1.

**Table 2:** Single factor variance analysis of Chinese residents’ media use.

Categories	All (*N* = 11,031, 100%)	Media use low-frequency group (*N*1 = 1,067, 9.7%)	Media use general (*N*2 = 7,415, 67.1%)	Media use high-frequency (*N*3 = 2,549, 23.2%)	*X^2^*	*P*
Gender					110.89	<0.001
Female	5,998 (54.4)	538 (50.4)	4,268 (57.6)	1,192 (46.8)		
Male	5,033 (45.6)	529 (49.6)	3,147 (42.4)	1,357 (53.2)		
Age (unit: years old)					219.27	<0.001
≤18	1,065 (9.7)	109 (10.2)	772 (10.4)	184 (7.2)		
19–40	5,332 (48.3)	257 (24.1)	3,829 (51.6)	1,246 (48.9)		
41–65	3,759 (34.1)	318 (29.8)	2,570 (34.7)	871 (34.2)		
≥66	875 (7.9)	383 (35.9)	244 (3.3)	248 (9.7)		
Highest educational level					96.77	<0.001
Primary school or below	1,127 (10.2)	453 (42.4)	481 (6.5)	193 (7.6)		
Junior middle school	3,417 (31.0)	340 (31.9)	2,334 (31.5)	743 (29.1)		
Technical secondary school or above	6,487 (58.8)	274 (25.7)	4,600 (62)	1,613 (63.3)		
Marital status					300.15	<0.001
Unmarried	4,363 (39.6)	263 (24.6)	3,115 (42.1)	985 (38.7)		
Married	6,226 (56.4)	658 (61.7)	4,089 (55.1)	1,479 (58.0)		
Divorced	207 (1.9)	14 (1.3)	142 (1.9)	51 (2.0)		
Widowed	235 (2.1)	132 (12.4)	69 (0.9)	34 (1.3)		
Whether to have children					169.17	<0.001
Without	5,062 (45.9)	306 (28.7)	3,600 (48.6)	1,156 (45.4)		
With	5,969 (54.1)	761 (71.3)	3,815 (51.4)	1,393 (54.6)		
Whether to have a regular occupation					47.32	0.009
Without	6,394 (58.0)	462 (43.3)	1,294 (17.5)	440 (17.3)		
With	4,637 (42.0)	605 (56.7)	6,121 (82.5)	2,109 (82.7)		
Whether to have debts					120.47	<0.001
Without	6,780 (61.5)	791 (74.1)	4,381 (59.1)	1,608 (63.1)		
With	4,251 (38.5)	276 (25.9)	3,034 (40.9)	941 (36.9)		
Whether to have medical insurance					210.71	<0.001
Without	2,299 (20.8)	224 (21.0)	1,507 (20.3)	568 (22.3)		
With	8,732 (79.2)	843 (79.0)	5,908 (79.7)	1981 (77.7)		
Whether to take medicine					147.69	<0.001
Without	8,996 (81.6)	703 (65.9)	6,283 (84.7)	2010 (78.9)		
With	2035 (18.4)	364 (34.1)	1,132 (15.3)	539 (21.1)		
Monthly *per capita* household income (yuan)					102.99	<0.001
≤6,000	7,500 (68.0)	861 (80.7)	5,061 (68.3)	1,578 (61.9)		
6,001–12,000	2,769 (25.1)	162 (15.2)	1886 (25.4)	721 (28.3)		
>12,000	762 (6.9)	44 (4.1)	468 (6.3)	250 (9.8)		
Pressure					4303.20	<0.001
Mild pressure	2,719 (24.6)	251 (23.5)	1946 (26.2)	522 (20.5)		
Moderate pressure	7,653 (69.4)	704 (66.0)	5,217 (70.4)	1732 (67.9)		
Major pressure	659 (6.0)	112 (10.5)	252 (3.4)	295 (11.6)		

### 3.2. Single factor variance analysis on Chinese residents’ media use

As shown in Table 2, a total of 11,031 valid questionnaires were collected in this survey. Among them, 5,998 (54.4%) were female, 1,065 (48.3%) were aged Less than 18 years, 6,487 (58.8%) were educated in technical college, and above, and 8,008 (72.6%) were urban residents.

**Figure 2:**
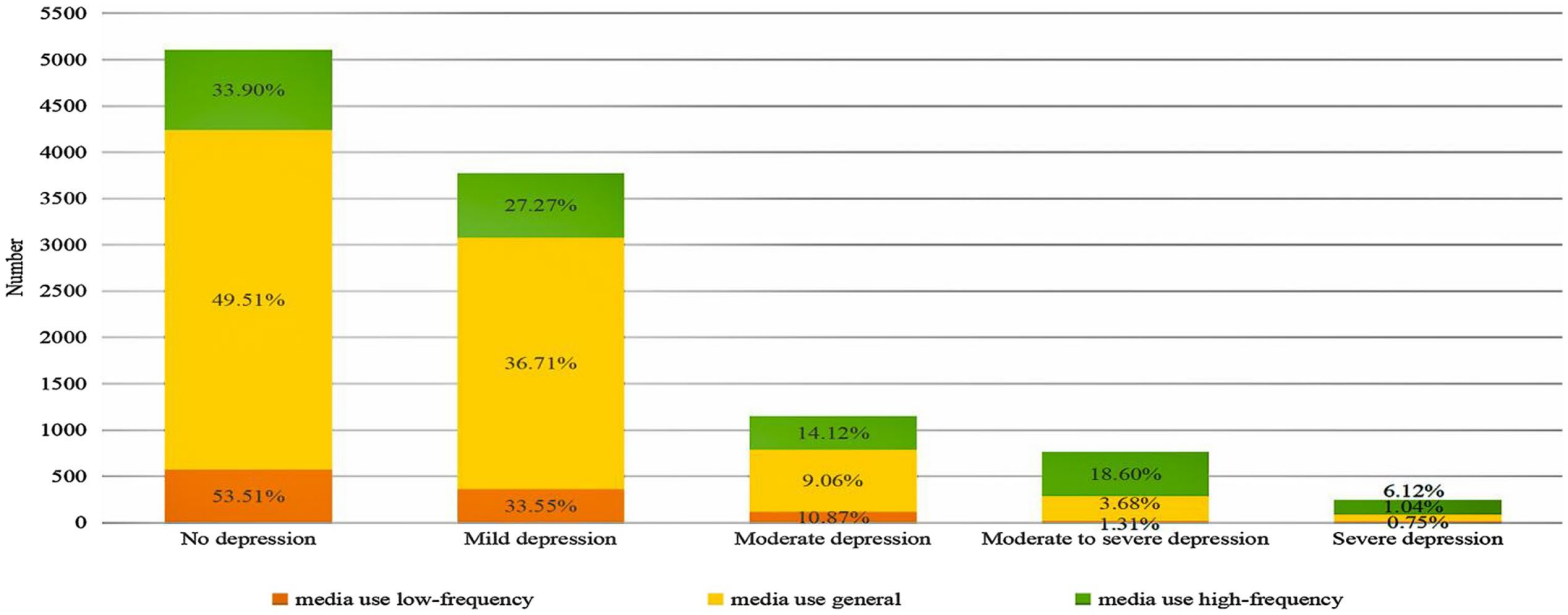
Number of residents concerned about depression and percentage of depression concern scores.

Through the potential profile analysis of media use, the most significant number of residents (51.6%) were aged 19–40 years in the media use general group, and the most significant number of people with a regular job (82.7%) were in the media use high-frequency group. Among the media use low-frequency group, more people (43.7%) were aged over 66 years than in other age groups. More people were married (61.7%) and had health insurance (79%). Mild pressure (26.2%) was prevalent among those with general media exposure, while severe pressure (11.6%) was more prevalent among those with media use high-frequency exposure.

The research showed that there was a statistically significant effect on gender, age, highest educational level, marital status, monthly *per capita* household earnings, whether to have children, whether to have a regular occupation, whether have medical insurance, whether to take medicine, debt, and pressure in depression (*p* < 0.05), indicating that all of these factors were influential factors in residents depression.

### 3.3. Media use and depression scores

The scores for all scales were shown in Table 3. Newspapers scoring the lowest (1.86 ± 1.08) and smartphones scoring the highest (4.33 ± 1.13), indicating that our residents preferred smart phone in terms of media use. The depression score of the participants in the study was moderate (6.18 ± 5.68), which indicated that Chinese residents did not know much about depression at present.

**Table 3:** The scores of media use and depression of the subjects.

Categories	Items	The range of scores	*M* ± SD
Newspaper	1	1–5	1.86 ± 1.08
Magazines	1	1–5	1.91 ± 1.05
Books	1	1–5	2.73 ± 1.26
Broadcast	1	1–5	2.10 ± 1.19
Television	1	1–5	3.24 ± 1.28
Personal computer	1	1–5	3.17 ± 1.44
Smart phone	1	1–5	4.33 ± 1.13
Depression	1	0–27	6.18 ± 5.68

### 3.4. Summary of resident depression scores

In the summary of residents’ depression scores (Figure 2), about 45.6% of residents were in the no depression stage. Only about 2.2% of residents were in the major depression stage.

Among the three categories of media-use groups, the media use general group has the highest score between no depression (3671), followed by the media use high-frequency group (864) and the media use low-frequency group (571). However, among their respective groups, the media use high-frequency group accounted for 33.9%, the media use general group for 49.51%, and the media use low-frequency group for 53.51%.

### 3.5. Regression analysis of predictive variables on depression

As shown in Table 4, depression scores were used as the dependent variable, and variables statistically significant in the univariate analysis were included as control variables in the linear regression model. Compared with media use general group, the analysis showed that in the media use low-frequency group (*β* = 0.019, *p* = 0.044) and the media use high-frequency group (*β* = 0.238, *p* < 0.001), it is significantly positively correlated with depression. The residents who had technical secondary school or above (*β* = 0.024, *p* = 0.018), aged 19–40 years (*β* = 0.044, *p* < 0.001), had to take medicine (*β* = 0.081, *p* < 0.001), had debt (*β* = 0.053, *p* < 0.001), had moderate pressure (*β* = 0.291, *p* < 0.001), had severe pressure (*β* = 0.288, *p* < 0.001) would have a higher risk of depression. The residents who aged over 66 years (*β* = −0.022, *p* = 0.023), married (*β* = −0.110, *p* < 0.001), had a regular occupation (*β* = −0.025, *p* = 0.008), had medical insurance (*β* = −0.063, *p* < 0.001) would have a lower risk of depression.

**Table 4:** Regression model of demographic and social factors and other scale scores to depression scores.

Model		Unstandardized coefficients		Standardized coefficients	*t*	*P*	EXP(β) 95% confidence interval	
		*B*	*SE*	*Beta*			LLCI	ULCI
Independent variable	Media use (Ref: general)							
	Low-frequency	0.363	0.180	0.019	2.018	0.044	0.010	0.715
	High-frequency	3.208	0.118	0.238	27.231	<0.001	2.977	3.439
Control variable	Highest educational level (Ref: primary school or below)							
	Technical secondary school or above	0.277	0.118	0.024	2.356	0.018	0.047	0.508
	Age (Ref: ≤ 18)							
	19–40	0.505	0.114	0.044	4.419	<0.001	0.281	0.729
	≥ 66	−0.468	0.206	−0.022	−2.275	0.023	−0.872	−0.065
	Marital status (Ref: unmarried)							
	Married	−1.260	0.108	−0.110	−11.709	<0.001	−1.472	−1.049
	Whether to have a regular occupation (Ref: without)							
	With	−0.355	0.134	−0.025	−2.646	0.008	−0.618	−0.092
	Whether to have medical insurance (Ref: without)							
	With	−0.887	0.123	−0.063	−7.201	<0.001	−1.128	−0.645
	Whether to take medicine (Ref: without)							
	With	1.193	0.135	0.081	8.804	<0.001	0.927	1.458
	Whether to have debts (Ref: without)							
	With	0.619	0.100	0.053	6.195	<0.001	0.423	0.814
	Pressure (Ref: mild pressure)							
	Moderate pressure	3.593	0.113	0.291	31.661	<0.001	3.371	3.815
	Major pressure	6.906	0.223	0.288	30.959	<0.001	6.469	7.343

As shown in Table 5, Among people in the media use low-frequency group, the residents who aged 19–40 years (*β* = 0.085, *p* = 0.006), the monthly household earnings *per capita* in the range of 6,001–12,000 yuan (*β* = 0.056, *p* = 0.047), had to take medicine (*β* = 0.096, *p* = 0.001), had debt (*β* = 0.098, *p* = 0.001), had moderate pressure (*β* = 0.274, *p* < 0.001), had severe pressure (*β* = 0.228, *p* < 0.001), and neuroticism (*β* = 0.102, *p* = 0.001) were higher would have a higher risk of depression, the residents’ agreeableness (*β* = −0.103, *p* = 0.001) were higher would have a lower risk of depression.

**Table 5:** Regression model analysis of depression in the media use low-frequency.

Model	Unstandardized coefficients		Standardized coefficients	*t*	*P*	EXP(β) 95% confidence interval	
	*B*	*SE*	*Beta*			LLCI	ULCI
Age (Ref: ≤18)							
19–40	1.062	0.387	0.085	2.741	0.006	0.302	1.821
*Per capita* monthly household income (Ref: ≤6,000)							
6,001–12,000	0.840	0.421	0.056	1.992	0.047	0.012	1.667
Whether to have debts (Ref: without)							
With	1.198	0.352	0.098	3.403	0.001	0.507	1.888
Whether to take medicine (Ref: without)							
With	1.084	0.341	0.096	3.183	0.001	0.416	1.752
Pressure (Ref: mild pressure)							
Moderate pressure	3.099	0.388	0.274	7.992	<0.001	2.338	3.860
Major pressure	3.983	0.595	0.228	6.692	<0.001	2.815	5.151
Big Five personality							
Neuroticism	0.417	0.124	0.102	3.353	0.001	0.173	0.661
Agreeableness	−0.401	0.117	−0.103	−3.432	0.001	−0.630	−0.172

As shown in Table 6, Among the residents in the media use general group, those who had technical secondary school or above (*β* = 0.037, *p* = 0.001), had to take medicine (*β* = 0.083, *p* < 0.001), had debt (*β* = 0.047, *p* < 0.001), had moderate pressure (*β* = 0.225, *p* < 0.001), had severe pressure (*β* = 0.214, *p* < 0.001), neuroticism (*β* = 0.159, *p* < 0.001) and openness (*β* = 0.047, *p* < 0.001) were higher would have a higher risk of depression.

**Table 6:** Regression model analysis of depression in the media use general.

Model	Unstandardized coefficients		Standardized coefficients	*t*	*P*	EXP(β) 95% confidence interval	
	*B*	*SE*	*Beta*			LLCI	ULCI
Highest educational level (Ref: primary school or below)							
Technical secondary school or above	0.363	0.109	0.037	3.334	0.001	0.149	0.576
Marital status (Ref: unmarried)							
Married	−0.687	0.112	−0.073	−6.109	<0.001	−0.908	−0.467
Whether to have medical insurance (Ref: without)							
With	−0.458	0.124	−0.039	−3.680	<0.001	−0.702	−0.214
Whether to take medicine (Ref: without)							
With	1.089	0.136	0.083	7.999	<0.001	0.822	1.356
Whether to have debts (Ref: Without)							
With	0.449	0.098	0.047	4.581	<0.001	0.257	0.641
Whether to have a regular occupation (Ref: without)							
With	−0.306	0.140	−0.025	−2.193	0.028	−0.580	−0.032
Pressure (Ref: mild pressure)							
Moderate pressure	2.317	0.114	0.225	20.272	<0.001	2.093	2.541
Major pressure	5.554	0.279	0.214	19.897	<0.001	5.007	6.101
Big Five personality							
Neuroticism	0.470	0.033	0.159	14.309	<0.001	0.406	0.535
Openness	0.137	0.032	0.047	4.328	<0.001	0.075	0.199
Conscientiousness	−0.294	0.032	−0.105	−9.095	<0.001	−0.357	−0.230
Extraversion	−0.184	0.030	−0.067	−6.187	<0.001	−0.242	−0.126
Agreeableness	−0.11	0.035	−0.035	−3.102	0.002	−0.179	−0.040
Social support							
Family support	−0.119	0.012	−0.114	−10.152	<0.001	−0.142	−0.096

As shown in Table 7, among the residents in the media use high-frequency group, those who were male (*β* = 0.036, *p* = 0.044), aged over 19 years (*β* = 0.051, *p* = 0.010), divorced (*β* = 0.044, *p* = 0.014), had to take medicine (*β* = 0.042, *p* = 0.024), and neuroticism was higher would have a higher risk of depression. The residents who married (*β* = −0.080, *p* < 0.001), had medical insurance (*β* = −0.085, *p* < 0.001), agreeableness (*β* = −0.107, *p* < 0.001), and conscientiousness (*β* = −0.116, *p* < 0.001) were higher would have a lower risk of depression.

**Table 7:** Regression model analysis of depression in the media use high-frequency.

Model	Unstandardized coefficients		Standardized coefficients	*t*	*P*	EXP (β) 95% confidence interval	
	*B*	*SE*	*Beta*			LLCI	ULCI
Gender (Ref: Female)							
Male	0.522	0.259	0.036	2.018	0.044	0.015	1.029
Age (Ref: ≤18)							
19–40	0.752	0.293	0.051	2.571	0.010	0.179	1.326
Highest educational level (Ref: Primary school or below)							
Technical secondary school or above	−0.796	0.296	−0.049	−2.687	0.007	−1.376	−0.215
Marital status (Ref: unmarried)							
Married	−1.190	0.293	−0.080	−4.067	<0.001	−1.764	−0.616
Divorced	2.304	0.935	0.044	2.466	0.014	0.472	4.137
Whether to have medical insurance (Ref: without)							
With	−1.495	0.316	−0.085	−4.730	<0.001	−2.115	−0.875
Whether to take medicine (Ref: without)							
With	0.752	0.333	0.042	2.261	0.024	0.100	1.405
Pressure (Ref: mild pressure)							
Moderate pressure	4.479	0.336	0.286	13.321	<0.001	3.820	5.139
Major pressure	7.938	0.483	0.348	16.444	<0.001	6.992	8.885
Social support							
Friend support	0.115	0.030	0.071	3.845	<0.001	0.056	0.173
Big Five personality							
Agreeableness	−0.528	0.101	−0.107	−5.239	<0.001	−0.726	−0.330
Conscientiousness	−0.577	0.099	−0.116	−5.854	<0.001	−0.771	−0.384
Neuroticism	0.391	0.108	0.069	3.623	<0.001	0.179	0.602

## 4. Discussion

According to the results of this study, compared with media use general group, both media use high-frequency and media use low-frequency groups are associated with depression. The higher or lower the degree of media use of the population, the more likely they are to be depressed. People with different degrees of media use have different influencing factors for depression.

The first finding of this study was that too much media use will lead to depression. Similar to this study, social media users who used it the most were three times more likely to become depressed than those who used it the least (Primack et al., 2021). The more time people spend on the media, the more depressed they feel (Shensa et al., 2017; Scherr, 2018; Yoon et al., 2019). A study on teenagers in Taiwan Province, China found that the more mobile phones teenagers use, the greater the risk of suicidal ideation (Yang et al., 2010). The reason may be that the more energy people spend on the media, the fewer opportunity they have for more valuable face-to-face interaction (Banjanin et al., 2015; Whaite et al., 2018), and the higher the risk of depression (Aydin et al., 2021). Furthermore, the internet contains mixed messages, and exposure to poor media can seriously reduce a person’s quality of life and increase their depression risk (Daly et al., 2010). According to some studies, teenagers can be harassed and victimized online, and cyberbullying is significantly and positively associated with depression (Hamm et al., 2015). Social media and smartphones are increasingly being misused, leading to loneliness, depression, and suicidal behavior (Twenge et al., 2018).

Another interesting finding of this study was that the media use low-frequency group was positively correlated with depression among the public. We speculate that people who use low-frequency media are mainly middle-aged or elderly people. It is common for middle-aged and older people to be unable to afford communication devices or not to be able to use video chat applications to communicate with others virtually (Seifert et al., 2021), resulting in lower media use, which in turn affects their social support and intergenerational relationships, and makes them more likely to suffer from depression (Wu and Chiou, 2020).

A similarity and a difference were also found in the factors influencing depression in the high-frequency, general, and blocked media-use groups. In these three groups of media use, debt, taking medicine, moderate and severe pressure, neuroticism of the Big Five personality traits, and agreeableness were common factors influencing depression. Only agreeableness negatively affects depression, and all the other influences positively impact it. Similarly, a Japanese study showed that older people who had debt were more likely to suffer from depression and that debt significantly and positively correlated with depression (Kaji et al., 2010). According to studies in Belgium, France, and Germany, there has also been a significant and positive association between financial debt and depression (Hiilamo and Grundy, 2018). People who perceive more pressure are more likely to develop depression (Dejonckheere et al., 2017). A positive relationship exists between early life pressures and the development of major depression in adulthood (LeMoult et al., 2020). High neuroticism is twice as likely to be depressed as low neuroticism, and agreeableness negatively affects depression (Nikcevic et al., 2021; Merrill et al., 2022).

There are also differences in the factors that impact depression among the three groups of media use. In the high-frequency media group, depression is associated with gender, age, social support of friends, and Big Five personality conscientiousness. Males between 19 and 40 and with friend support are more likely to be depressed, while conscientious personalities are less likely to be depressed. According to Kotov, conscientiousness may be negatively associated with depression (Kotov et al., 2010). The risk of depression increases with age (Kaji et al., 2010). Some studies are similar to the present study, but there are also some that conflict with it. It has been found that depression is less likely to affect people who have friend support. It was negatively associated with depression to have friend support (Chang et al., 2018). There were twice as many women as men who were depressed (Brody et al., 2018). According to the media use general group, education, openness, extraversion of the Big Five personality, and family support are factors that contribute to depression. Residents’ higher education degrees and openness are associated with a higher risk of depression. Comparatively, those with higher levels of extraversion and family support are less likely to suffer from depression. In contrast to the present study, Nikčević’s findings suggest that openness to personality may hinder depression (Nikcevic et al., 2021). The results of previous studies were also consistent with the findings of these factors (Bum and Jeon, 2016; Watson et al., 2019; Guo et al., 2021). Age and monthly income *per capita* influence depression in the media use low-frequency group. Accordingly, residents aged 19–40 with a monthly income between 6,001–12,000 yuan are more likely to suffer from depression. It has been shown that middle-aged people are more likely to suffer depression (Liu et al., 2020). Contrary to this study, higher and middle-income levels were associated with a lower risk of depression (Tay et al., 2022).

## 5. Conclusion

According to the study, Chinese residents are divided into three categories based on their media use: media use low-frequency group, media use general group, and media use high-frequency group. Second, we examined the relationship between media use and depression among people with different degrees of media consumption. Compared with media use general group, there is a positive correlation between media use high-frequency group and media use low-frequency group. This study identified the influencing factors of depression among people with different degrees of media use.

In this study, first of all, we should make strategies according to the different degrees of media exposure. For general and high-frequency media users, the government can encourage them to engage in appropriate physical activities and relevant organizations to carry out more popular science and services on mental health to guide the public to use the media reasonably and moderately. Media users of low-frequency can be enabled to utilize the media correctly or learn the valuable functions of the media. Secondly, more accurate strategies should be made according to the influencing factors of these three degrees of media users. In the high-frequency media group, we found that male residents aged between 19 and 40 with friends to support them have an increased risk of depression. Therefore, it is suggested that the same age groups should pay more attention to the men in this age group to enhance their sense of security from friends and avoid depression. According to the media use general group, “residents with a college degree or higher and an openness in the Big 5 personality were at increased risk of depression.” Thus, it is suggested that the government adopt different guidelines for residents with varying personality characteristics to promote accurate mental health communication. In the media use low-frequency group, we found that “residents aged 19–40 with a *per capita* monthly income of 6,001 yuan have a higher risk of depression.” This phenomenon shows that young and middle-aged people’s participation in appropriate physical activities benefits their physical and mental health.

## 6. Highlights and limitations of research

It is the first large-scale study of depression in the Chinese mainland with a representative sample size. The study is the first to examine depression with media use as a variable and a potential population profile, which is at least somewhat innovative.

There are some limitations to this study: firstly, our study data took the form of self-reporting, which makes it challenging to eliminate recall bias; Secondly, this study used cross-sectional data as the data source, which makes it challenging to infer the causal relationship. Third, due to the limitations of sampling methods, there may be bias in sample selection.

## Data availability statement

The raw data supporting the conclusions of this article will be made available by the authors, without undue reservation.

## Ethics statement

The studies involving human participants were reviewed and approved by the Institutional Review Committee of Jinan University, Guangzhou, China (JNUKY-2021-018). All methods were performed in accordance with relevant guidelines and regulations.

## Author contributions

FG, PY, ZM, and LZ: conceptualization. FG, PY, LY, and SF: methodology. ZM, GG, and LZ: investigation. PY and YJ: visualization. FG and ZM: supervision. FG and PY: writing-original draft preparation. FG, PY, LZ, YL, LY, and ZM: writing-review and editing. All authors contributed to the article and approved the submitted version.

## Funding

This study was supported by National Social Science Foundation Project in 2019: Study on the Media Philosophy of the North American School of Media Environment (Project No. 19BZX036) and the Cultural Research Center of Countries along the “Southwest Silk Road” of the Ethnic Affairs Commission of the People’s Republic of China (Jishou University).

## Conflict of interest

The authors declare that the research was conducted in the absence of any commercial or financial relationships that could be construed as a potential conflict of interest.

## Publisher’s note

All claims expressed in this article are solely those of the authors and do not necessarily represent those of their affiliated organizations, or those of the publisher, the editors and the reviewers. Any product that may be evaluated in this article, or claim that may be made by its manufacturer, is not guaranteed or endorsed by the publisher.

## References

[ref1] Alsubaie M. M. Stain H. J. Webster L. A. D. Wadman R. (2019). The role of sources of social support on depression and quality of life for university students. Int. J. Adolesc. Youth 24, 484–496. doi: 10.1080/02673843.2019.1568887

[ref2] Andersen I. Thielen K. Bech P. Nygaard E. Diderichsen F. (2011). Increasing prevalence of depression from 2000 to 2006. Scand. J. Public Health 39, 857–863. doi: 10.1177/1403494811424611 21965477

[ref3] Assari S. (2017). Social determinants of depression: the intersections of race, gender, and socioeconomic status. Brain Sci. 7:156. doi: 10.3390/brainsci7120156, PMID: 29186800 PMC5742759

[ref4] Aydin S. Kocak O. Shaw T. A. Buber B. Akpinar E. Z. Younis M. Z. (2021). Investigation of the effect of social media addiction on adults with depression. Healthcare (Basel) 9:450. doi: 10.3390/healthcare9040450, PMID: 33920478 PMC8070133

[ref5] Banjanin N. Banjanin N. Dimitrijevic I. Pantic I. (2015). Relationship between internet use and depression: focus on physiological mood oscillations, social networking and online addictive behavior. Comput. Hum. Behav. 43, 308–312. doi: 10.1016/j.chb.2014.11.013

[ref6] Bickham D. S. Hswen Y. Rich M. (2015). Media use and depression: exposure, household rules, and symptoms among young adolescents in the USA. Int. J. Public Health 60, 147–155. doi: 10.1007/s00038-014-0647-6, PMID: 25586816 PMC4375733

[ref7] Blease C. R. (2015). Too many friends, too few likes? Evolutionary psychology and ‘Facebook depression’. Rev. Gen. Psychol. 19, 1–13. doi: 10.1037/gpr0000030

[ref8] Brody D. J. Pratt L. A. Hughes J. P.. (2018). *Prevalence of Depression among Adults Aged 20 and over: United States, 2013-2016*. Available at: https://www.cdc.gov/nchs/products/databriefs/db303.htm. (Accessed August 5, 2022). 29638213

[ref9] Bukhari S. R. Afzal F. (2017). Perceived social support predicts psychological problems among university students. Int. J. Indian Psychol. 4, 18–27. doi: 10.25215/0402.082

[ref10] Bum C.-H. Jeon I.-K. (2016). Structural relationships between students' social support and self-esteem, depression, and happiness. Soc. Behav. Personal. Int. J. 44, 1761–1774. doi: 10.2224/sbp.2016.44.11.1761

[ref11] Cellini N. Canale N. Mioni G. Costa S. (2020). Changes in sleep pattern, sense of time and digital media use during COVID-19 lockdown in Italy. J. Sleep Res. 29:e13074. doi: 10.1111/jsr.13074, PMID: 32410272 PMC7235482

[ref12] Chang C.-W. Yuan R. Chen J.-K. (2018). Social support and depression among Chinese adolescents: the mediating roles of self-esteem and self-efficacy. Child Youth Serv. Rev. 88, 128–134. doi: 10.1016/j.childyouth.2018.03.001

[ref13] Chen F. Zheng D. Liu J. Gong Y. Guan Z. Lou D. (2020). Depression and anxiety among adolescents during COVID-19: a cross-sectional study. Brain Behav. Immun. 88, 36–38. doi: 10.1016/j.bbi.2020.05.061, PMID: 32464156 PMC7247496

[ref14] Daly E. J. Trivedi M. H. Wisniewski S. R. Nierenberg A. A. Gaynes B. N. Warden D.. (2010). Health-related quality of life in depression: a STAR*D report. Ann. Clin. Psychiatry 22, 43–55. doi: 10.1038/415276a 20196982

[ref15] Dejonckheere E. Bastian B. Fried E. I. Murphy S. C. Kuppens P. (2017). Perceiving social pressure not to feel negative predicts depressive symptoms in daily life. Depress. Anxiety 34, 836–844. doi: 10.1002/da.22653 28499066

[ref16] Den Hamer A. H. Konijn E. A. Plaisier X. S. Keijer M. G. Krabbendam L. C. Bushman B. J. (2017). The content-based media exposure scale (C-ME): development and validation. Comput. Hum. Behav. 72, 549–557. doi: 10.1016/j.chb.2017.02.050

[ref17] Fleming T. M. Clark T. Denny S. Bullen P. Crengle S. Peiris-John R.. (2014). Stability and change in the mental health of New Zealand secondary school students 2007-2012: results from the national adolescent health surveys. Aust. N. Z. J. Psychiatry 48, 472–480. doi: 10.1177/0004867413514489, PMID: 24317154

[ref18] Frederick D. A. Daniels E. A. Bates M. E. Tylka T. L. (2017). Exposure to thin-ideal media affect most, but not all, women: results from the perceived effects of media exposure scale and open-ended responses. Body Image 23, 188–205. doi: 10.1016/j.bodyim.2017.10.006 29132044

[ref19] Frison E. Eggermont S. (2016). Exploring the relationships between different types of Facebook use, perceived online social support, and adolescents' depressed mood. Soc. Sci. Comput. Rev. 34, 153–171. doi: 10.1177/0894439314567449

[ref20] Frost R. L. Rickwood D. J. (2017). A systematic review of the mental health outcomes associated with Facebook use. Comput. Hum. Behav. 76, 576–600. doi: 10.1016/j.chb.2017.08.001

[ref21] Fujise N. Abe Y. Fukunaga R. Nakagawa Y. Nishi Y. Koyama A.. (2016). Comparisons of prevalence and related factors of depression in middle-aged adults between urban and rural populations in Japan. J. Affect. Disord. 190, 772–776. doi: 10.1016/j.jad.2015.11.020, PMID: 26618770

[ref22] Gosling S. D. Rentfrow P. J. Swann W. B. (2003). A very brief measure of the big-five personality domains. J. Res. Pers. 37, 504–528. doi: 10.1016/s0092-6566(03)00046-1

[ref23] Guo L. Fan H. Xu Z. Li J. Chen T. Zhang Z.. (2021). Prevalence and changes in depressive symptoms among postgraduate students: a systematic review and meta-analysis from 1980 to 2020. Stress. Health 37, 835–847. doi: 10.1002/smi.3045, PMID: 33871902

[ref24] Hamm M. P. Newton A. S. Chisholm A. Shulhan J. Milne A. Sundar P.. (2015). Prevalence and effect of cyberbullying on children and young people: a scoping review of social media studies. JAMA Pediatr. 169, 770–777. doi: 10.1001/jamapediatrics.2015.0944, PMID: 26098362

[ref25] He F. Zhou Q. Li J. Cao R. Guan H. (2014). Effect of social support on depression of internet addicts and the mediating role of loneliness. Int. J. Ment. Health Syst. 8:34. doi: 10.1186/1752-4458-8-34, PMID: 25147581 PMC4139580

[ref26] Hiilamo A. Grundy E. (2018). Household debt and depressive symptoms among older adults in three continental European countries. Ageing Soc. 40, 412–438. doi: 10.1017/s0144686x18001113

[ref27] Holfeld B. Sukhawathanakul P. (2017). Associations between internet attachment, cyber victimization, and internalizing symptoms among adolescents. Cyberpsychol. Behav. Soc. Netw. 20, 91–96. doi: 10.1089/cyber.2016.0194, PMID: 28080133

[ref28] Holling H. Schlack R. Petermann F. Ravens-Sieberer U. Mauz E. KiGGS Study Group (2014). Psychopathological problems and psychosocial impairment in children and adolescents aged 3-17 years in the German population: prevalence and time trends at two measurement points (2003-2006 and 2009-2012): results of the KiGGS study: first follow-up (KiGGS wave 1). Bundesgesundheitsblatt Gesundheitsforschung Gesundheitsschutz 57, 807–819. doi: 10.1007/s00103-014-1979-3, PMID: 24950830

[ref29] Jelenchick L. A. Eickhoff J. C. Moreno M. A. (2013). Facebook depression? Social networking site use and depression in older adolescents. J. Adolesc. Health 52, 128–130. doi: 10.1016/j.jadohealth.2012.05.008, PMID: 23260846

[ref30] Kaji T. Mishima K. Kitamura S. Enomoto M. Nagase Y. Li L.. (2010). Relationship between late-life depression and life stressors: large-scale cross-sectional study of a representative sample of the Japanese general population. Psychiatry Clin. Neurosci. 64, 426–434. doi: 10.1111/j.1440-1819.2010.02097.x, PMID: 20492557

[ref31] Kayiş A. R. Satici S. A. Yilmaz M. F. Şimşek D. Ceyhan E. Bakioğlu F. (2016). Big five-personality trait and internet addiction: a meta-analytic review. Comput. Hum. Behav. 63, 35–40. doi: 10.1016/j.chb.2016.05.012

[ref32] Koc M. Gulyagci S. (2013). Facebook addiction among Turkish college students: the role of psychological health, demographic, and usage characteristics. Cyberpsychol. Behav. Soc. Netw. 16, 279–284. doi: 10.1089/cyber.2012.0249, PMID: 23286695

[ref33] Kotov R. Gamez W. Schmidt F. Watson D. (2010). Linking "big" personality traits to anxiety, depressive, and substance use disorders: a meta-analysis. Psychol. Bull. 136, 768–821. doi: 10.1037/a0020327, PMID: 20804236

[ref34] Kross E. Verduyn P. Demiralp E. Park J. Lee D. S. Lin N.. (2013). Facebook use predicts declines in subjective well-being in young adults. PLoS One 8:e69841. doi: 10.1371/journal.pone.0069841, PMID: 23967061 PMC3743827

[ref35] LeMoult J. Humphreys K. L. Tracy A. Hoffmeister J. A. Ip E. Gotlib I. H. (2020). Meta-analysis: exposure to early life stress and risk for depression in childhood and adolescence. J. Am. Acad. Child Adolesc. Psychiatry 59, 842–855. doi: 10.1016/j.jaac.2019.10.011, PMID: 31676392 PMC11826385

[ref36] Lepine J. P. Briley M. (2011). The increasing burden of depression. Neuropsychiatr. Dis. Treat. 7, 3–7. doi: 10.2147/NDT.S19617, PMID: 21750622 PMC3131101

[ref37] Liu C. Liu D. Huang N. Fu M. Ahmed J. F. Zhang Y.. (2020). The combined impact of gender and age on post-traumatic stress symptoms, depression, and insomnia during COVID-19 outbreak in China. Front. Public Health 8:620023. doi: 10.3389/fpubh.2020.620023, PMID: 33553099 PMC7859527

[ref38] Liu M. Ming Q. Yi J. Wang X. Yao S. (2016). Screen time on school days and risks for psychiatric symptoms and self-harm in mainland Chinese adolescents. Front. Psychol. 7:574. doi: 10.3389/fpsyg.2016.00574, PMID: 27199811 PMC4842926

[ref39] Löwe B. Kroenke K. Herzog W. Gräfe K. (2004). Measuring depression outcome with a brief self-report instrument: sensitivity to change of the patient health questionnaire (PHQ-9). J. Affect. Disord. 81, 61–66. doi: 10.1016/s0165-0327(03)00198-8, PMID: 15183601

[ref40] Merrill R. A. Cao C. Primack B. A. (2022). Associations between social media use, personality structure, and development of depression. J. Affect. Disord. Rep. 10:100385. doi: 10.1016/j.jadr.2022.100385

[ref41] Meshi D. Ellithorpe M. E. (2021). Problematic social media use and social support received in real-life versus on social media: associations with depression, anxiety and social isolation. Addict. Behav. 119:106949. doi: 10.1016/j.addbeh.2021.106949, PMID: 33934007

[ref42] Neese A. L. Pittman L. D. Hunemorder R. (2013). Depressive symptoms and somatic complaints among Zambian adolescents: associations with stress and coping. J. Res. Adolesc. 23, 118–127. doi: 10.1111/j.1532-7795.2012.00834.x

[ref43] Nesi J. Miller A. B. Prinstein M. J. (2017). Adolescents' depressive symptoms and subsequent technology-based interpersonal behaviors: a multi-wave study. J. Appl. Dev. Psychol. 51, 12–19. doi: 10.1016/j.appdev.2017.02.002, PMID: 28966426 PMC5619675

[ref44] Nikcevic A. V. Marino C. Kolubinski D. C. Leach D. Spada M. M. (2021). Modelling the contribution of the big five personality traits, health anxiety, and COVID-19 psychological distress to generalised anxiety and depressive symptoms during the COVID-19 pandemic. J. Affect. Disord. 279, 578–584. doi: 10.1016/j.jad.2020.10.053, PMID: 33152562 PMC7598311

[ref45] Nimrod G. (2013). Online depression communities: members' interests and perceived benefits. Health Commun. 28, 425–434. doi: 10.1080/10410236.2012.691068, PMID: 22809441

[ref46] Petersen A. (2001). Biofantasies: genetics and medicine in the print news media. Soc. Sci. Med. 52, 1255–1268. doi: 10.1016/s0277-9536(00)00229-x 11281408

[ref47] Pfeil U. Arjan R. Zaphiris P. (2009). Age differences in online social networking–a study of user profiles and the social capital divide among teenagers and older users in MySpace. Comput. Hum. Behav. 25, 643–654. doi: 10.1016/j.chb.2008.08.015

[ref48] Primack B. A. Shensa A. Sidani J. E. Escobar-Viera C. G. Fine M. J. (2021). Temporal associations between social media use and depression. Am. J. Prev. Med. 60, 179–188. doi: 10.1016/j.amepre.2020.09.014, PMID: 33309454 PMC8261713

[ref49] Przepiorka A. Blachnio A. Cudo A. (2021). Relationships between morningness, big five personality traits, and problematic internet use in young adult university students: mediating role of depression. Chronobiol. Int. 38, 248–259. doi: 10.1080/07420528.2020.1851703, PMID: 33317359

[ref50] Ranjan L. K. Gupta P. R. Srivastava M. Gujar N. M. (2021). Problematic internet use and its association with anxiety among undergraduate students. Asian J. Soc. Health Behav. 4:137. doi: 10.4103/shb.shb-30-21

[ref51] Rodriguez-Hidalgo A. J. Pantaleon Y. Dios I. Falla D. (2020). Fear of COVID-19, stress, and anxiety in university undergraduate students: a predictive model for depression. Front. Psychol. 11:591797. doi: 10.3389/fpsyg.2020.591797, PMID: 33224080 PMC7674167

[ref52] Scherr S. (2018). Traditional media use and depression in the general population: evidence for a non-linear relationship. Curr. Psychol. 40, 957–972. doi: 10.1007/s12144-018-0020-7

[ref53] Schou Andreassen C. Billieux J. Griffiths M. D. Kuss D. J. Demetrovics Z. Mazzoni E.. (2016). The relationship between addictive use of social media and video games and symptoms of psychiatric disorders: a large-scale cross-sectional study. Psychol. Addict. Behav. 30, 252–262. doi: 10.1037/adb0000160, PMID: 26999354

[ref54] Seabrook E. M. Kern M. L. Rickard N. S. (2016). Social networking sites, depression, and anxiety: a systematic review. JMIR Ment. Health 3:e50. doi: 10.2196/mental.5842, PMID: 27881357 PMC5143470

[ref55] Seale C. (2003). Health and media: an overview. Sociol. Health Illn. 25, 513–531. doi: 10.1111/1467-9566.t01-1-00356 12919443

[ref56] Seifert A. Cotten S. R. Xie B. (2021). A double burden of exclusion? Digital and social exclusion of older adults in times of COVID-19. J. Gerontol. B Psychol. Sci. Soc. Sci. 76, e99–e103. doi: 10.1093/geronb/gbaa098, PMID: 32672332 PMC7454901

[ref57] Shensa A. Escobar-Viera C. G. Sidani J. E. Bowman N. D. Marshal M. P. Primack B. A. (2017). Problematic social media use and depressive symptoms among U.S. young adults: a nationally-representative study. Soc. Sci. Med. 182, 150–157. doi: 10.1016/j.socscimed.2017.03.061, PMID: 28446367 PMC5476225

[ref58] Shi L. Que J. Y. Lu Z. A. Gong Y. M. Liu L. Wang Y. H.. (2021). Prevalence and correlates of suicidal ideation among the general population in China during the COVID-19 pandemic. Eur. Psychiatry 64:e18. doi: 10.1192/j.eurpsy.2021.5, PMID: 33686933 PMC7943957

[ref59] Steers M.-L. N. Wickham R. E. Acitelli L. K. (2014). Seeing everyone Else's highlight reels: how Facebook usage is linked to depressive symptoms. J. Soc. Clin. Psychol. 33, 701–731. doi: 10.1521/jscp.2014.33.8.701

[ref60] Tay W. W. Y. Jesuthasan J. Wan K. S. Ong T. Mustapha F. (2022). Eighteen months into the COVID-19 pandemic: the prevalence of depression, anxiety, and stress symptoms in Southeast Asia and the associated demographic factors. Front. Public Health 10:863323. doi: 10.3389/fpubh.2022.863323, PMID: 35991032 PMC9387355

[ref61] Twenge J. M. Joiner T. E. Rogers M. L. Martin G. N. (2018). Increases in depressive symptoms, suicide-related outcomes, and suicide rates among US adolescents after 2010 and links to increased new media screen time. Clin. Psychol. Sci. 6, 3–17. doi: 10.1177/2167702617723376

[ref62] Von Soest T. Wichstrom L. (2014). Secular trends in depressive symptoms among Norwegian adolescents from 1992 to 2010. J. Abnorm. Child Psychol. 42, 403–415. doi: 10.1007/s10802-013-9785-1, PMID: 23888312

[ref63] Watson D. Stanton K. Khoo S. Ellickson-Larew S. Stasik-O'Brien S. M. (2019). Extraversion and psychopathology: a multilevel hierarchical review. J. Res. Pers. 81, 1–10. doi: 10.1016/j.jrp.2019.04.009

[ref64] Weiß M. Baumeister H. Cohrdes C. Deckert J. Gründahl M. Pryss R.. (2022). Extraversion moderates the relationship between social media use and depression. J. Affect. Disord. Rep. 8:100343. doi: 10.1016/j.jadr.2022.100343

[ref65] Whaite E. O. Shensa A. Sidani J. E. Colditz J. B. Primack B. A. (2018). Social media use, personality characteristics, and social isolation among young adults in the United States. Personal. Individ. Differ. 124, 45–50. doi: 10.1016/j.paid.2017.10.030 PMC13309234 42367331

[ref66] World Health Organization (2016). *Depression*. World Health Organization. Available: at: https://www.who.int/newsroom/fact-sheets/detail/depression/. (Accessed August 2022).

[ref67] Wu H. Y. Chiou A. F. (2020). Social media usage, social support, intergenerational relationships, and depressive symptoms among older adults. Geriatr. Nurs. 41, 615–621. doi: 10.1016/j.gerinurse.2020.03.016, PMID: 32268948

[ref68] Yang Y. S. Yen J. Y. Ko C. H. Cheng C. P. Yen C. F. (2010). The association between problematic cellular phone use and risky behaviors and low self-esteem among Taiwanese adolescents. BMC Public Health 10:217. doi: 10.1186/1471-2458-10-217, PMID: 20426807 PMC2873584

[ref69] Yoon S. Kleinman M. Mertz J. Brannick M. (2019). Is social network site usage related to depression? A meta-analysis of Facebook-depression relations. J. Affect. Disord. 248, 65–72. doi: 10.1016/j.jad.2019.01.026, PMID: 30711871

[ref70] Zhang X. Zhang X. Li Y. Chen T. Siow L. Ye X.. (2022). What are the acceptances and associated influences of hospice care in mainland China? A national cross-sectional study. Front. Public Health 10:985218. doi: 10.3389/fpubh.2022.985218, PMID: 36211671 PMC9544594

[ref71] Zhong B. Huang Y. Liu Q. (2021). Mental health toll from the coronavirus: social media usage reveals Wuhan residents' depression and secondary trauma in the COVID-19 outbreak. Comput. Human Behav. 114:106524. doi: 10.1016/j.chb.2020.106524, PMID: 32836728 PMC7428783

[ref72] Zimet G. D. Powell S. S. Farley G. K. Werkman S. Berkoff K. A. (1990). Psychometric characteristics of the multidimensional scale of perceived social support. J. Pers. Assess. 55, 610–617. doi: 10.1080/00223891.1990.9674095 2280326

